# An Efficient Interface for the Integration of IoT Devices with Smart Grids

**DOI:** 10.3390/s20102849

**Published:** 2020-05-17

**Authors:** Felipe Viel, Luis Augusto Silva, Valderi Reis Quietinho Leithardt, Juan Francisco De Paz Santana, Raimundo Celeste Ghizoni Teive, Cesar Albenes Zeferino

**Affiliations:** 1 Laboratory of Embedded and Distribution Systems, University of Vale do Itajaí, Rua Uruguai 458, C.P. 360, Itajaí 88302-901, Brazil; viel@univali.br (F.V.); luis.silva@edu.univali.br (L.A.S.); zeferino@univali.br (C.A.Z.); 2 Departamento de Informática, Universidade da Beira Interior, 6201-001 Covilhã, Portugal; 3 COPELABS, Universidade Lusófona de Humanidades e Tecnologias, 1749-024 Lisboa, Portugal; 4 Instituto de Telecomunicações, Universidade da Beira Interior, 6201-001 Covilhã, Portugal; 5 Expert Systems and Applications Lab, Faculty of Science, University of Salamanca, Plaza de los Caídos s/n, 37008 Salamanca, Spain; fcofds@usal.es; 6 Laboratory of Applied Intelligence, University of Vale do Itajaí, Rua Uruguai 458, C.P. 360, Itajaí 88302-901, Brazil; rteive@univali.br

**Keywords:** internet of things, smart grids, communication protocol, interoperability, CoAP, OSGP

## Abstract

The evolution of computing devices and ubiquitous computing has led to the development of the Internet of Things (IoT). Smart Grids (SGs) stand out among the many applications of IoT and comprise several embedded intelligent technologies to improve the reliability and the safety of power grids. SGs use communication protocols for information exchange, such as the Open Smart Grid Protocol (OSGP). However, OSGP does not support the integration with devices compliant with the Constrained Application Protocol (CoAP), a communication protocol used in conventional IoT systems. In this sense, this article presents an efficient software interface that provides integration between OSGP and CoAP. The results obtained demonstrate the effectiveness of the proposed solution, which presents low communication overhead and enables the integration between IoT and SG systems.

## 1. Introduction

The evolution of computing devices and ubiquitous computing has led to the development of the Internet of Things (IoT). This technology encompasses several devices with different functionalities that can be interconnected in the same environment or even in separate environments. IoT applications are diverse, and some typical applications include Industry 4.0, logistics, and smart cities [1]. The latter is further subdivided into traffic, sanitation, agriculture, security surveillance, and Smart Grids (SGs), which are the focus of this article.

SGs are different from traditional power grids, which are structures with the sole function of transmitting and distributing electricity from remote plants to end consumers. On the other hand, SGs make intensive use of communication technologies in the power grid to enable the transfer of status information from various components in the grid. This feature allows to implement strategies for system operation and control more efficiently than conventional solutions. SGs, in conjunction with IoT, can be seen as an answer to fundamental questions in the energy market. In fact, what makes an SG “smart” is its ability to bidirectional communication, sensing, management possibilities, and the use of protocols that allow data exchange, as seen in [2,3,4,5,6]. Consequently, all stakeholders in the electricity sector, from generation to industrial or residential users, must work together for the continuous flow of generation and use of information generated by SG. With that, the integration between the different protocols of IoT and SG is the main challenge caused by the development directed to different purposes.

As highlighted in [7], the communication architecture is a quite important aspect to be considered in SGs, because this architecture must provide the necessary infrastructure to support the expected SG functionalities based on automated and intelligent management and control functions in electrical power systems. Besides, it must fulfill the performance requirements, including timing, since timing is a critical aspect to take into account in SGs communication, especially with regards to power system operation and protection.

Cyber-Physical Systems (CPSs), such as SG and IoT systems, are represented by platforms that are integrated through connectivity protocols that enable to share information among the different devices [8,9]. Several standards can be used for IoT communication, such as Constrained Application Protocol (CoAP) and Message Queue Telemetry Transport (MQTT). These protocols can be applied to a general IoT context, but are not recommended or standardized for use in SGs, as the Open Smart Grid Protocol (OSGP) is. A solution is necessary to integrate those protocols and enable data exchanges between general application protocols and specific application protocols such as OSGP.

Given the heterogeneity of general and specific applications and protocols, we identified a gap regarding the mapping of data packets between CoAP and OSGP. This mapping would enable IoT systems applied to residential and industrial plants to obtain and provide information to SGs. Within this context, this paper presents the integration between the CoAP and OSGP communication protocols in a solution that we call CoAP and OSGP Integration for the Internet of Things (COIIoT). To the best of our knowledge, based on the literature systematic mapping executed, this is the first adaptation between CoAP and OSGP.

The remainder of this article is organized as follows. Section 2 provides a brief background on communication protocols with an emphasis on CoAP and OSGP. Next, Section 3 discusses some related work. Section 4, in turn, presents the architecture of the proposed integration architecture. Following, Section 5 presents the materials and methods employed and discusses the results obtained in the experiments. Finally, in Section 6, we give the final remarks and discuss future work.

## 2. Background

This section extends the contextualization above and presents background on SGs and IoT communication protocols.

### 2.1. Smart Grids

SGs introduced a new paradigm for electrical power systems, and this technology is being developed in order to meet the rising electricity requirement [10]. This concept intrinsically carries a set of features, including efficiency, intelligence, quality, accommodation, reliability, green, and resilience [11]. SGs are complex CPSs that take the benefit of several embedded intelligent technologies to improve the reliability and the safety of power grids.

The main changes with regards to the implementation of SGs [12]: the use of smart meters, the increase of information provided to the consumers, the possibility of consumers to supply energy (renewable and intermittent energy sources) to the grid, the impact of electric vehicles as generating and storage batteries to the reliability of the distribution networks, and the need for extensive use of technologies to increase the safety and reliability of the electrical distribution systems. Furthermore, SGs have some particular features [11], which include: (i) self-healing capability, which means the distribution network has both self-diagnosis and self-decision abilities; (ii) the consumer becomes empowered, not only because he/she will be a continuous source of information and data, but also will be able to receive information and commands from the electrical utility when participating in a demand response program; (iii) tolerant to physical and cyber-attacks; as well as (iv) to provide quality of energy to the consumers. It is also necessary to accommodate a wide variety of renewable energy sources (distributed generation) in the SGs to reduce the environmental impact of generation systems, and to enable the competitive energy markets.

It is important to emphasize that communication technologies and network security issues will play a crucial role in the SGs. This role is due mainly to some intrinsic characteristics of these grids: a large number of electronic meters, sensors, electric vehicles, control and automation devices, and distributed generation. Besides, the implementation of self-healing actions in the SGs depend on communication quality [13]. Concerning the cybersecurity, in [14], the authors pointed up the need for this service, enabling the electrical network to identify, react to attacks, and prevent failures in real-time.

The consumer empowerment in SGs is also emphasized in [5,15]. In [5], the author comments that this empowerment is due mainly to the Advanced Metering Infrastructure (AMI), which is used to measure, acquire, and analyze the energy consumption data in order to supply information for each consumer. This process involves both bidirectional and cost-efficient communication. On the other hand, in [15], is presented the term prosumer for representing consumer importance, and he has an active role in the SG context. This fact can be observed, especially concerning the microgrids

In [6], the authors tackle the aspects to be observed in the communication architecture design for SGs, considering the presence of renewable energy sources. In this case, according to [6], three aspects must be taken into account: Feasibility: possibility of having different protocol standards and IP address mechanism in the grid; Scalability: it refers to the data flow from distributed generation as well as the transmission systems; Reliability: it means the definition of some performance metrics is necessary, especially concerning latency and packet loss rate. Then, the importance of communication infrastructure and its technologies, including communication protocols, is quite evident in the SGs context.

### 2.2. IoT Communication Protocols

In IoT, communication devices and networks are not isolated but connected and integrated to form a computer network. This feature brings the need for regular communication within the computer network, but it is constrained by the devices that make up IoT systems, which has limited power supply and storage and processing capacities.

Traditional protocols, such as Transmission Control Protocol (TCP), do not deal well with the limitations imposed by IoT devices, given the overheads generated by the process layers in these protocols. Furthermore, these protocols face addressing issues with each device. One of the alternatives to solve this problem was the adoption of Internet Protocol v6 (IPv6) and its new concepts, such as IPv6 over Low power Wireless Personal Area Networks (6LoWPAN), conceived of the idea of assigning an address to computationally restricted devices [16]. To this end, the Internet Engineering Task Force (IETF) and other standards bodies have defined and developed application protocols for resource-constrained devices. Examples of protocols developed by IETF are CoAP and Routing Protocol for Low Power and Lossy Networks (RPL) [17].

In addition to CoAP, other protocols are used to develop solutions for IoT, especially when focusing on Machine-to-Machine (M2M) communication. These alternatives may include: (i) MQTT; (ii) Extensible Messaging and Presence Protocol (XMPP); (iii) RESTful Services; (iv) Advanced Message Queuing Protocol (AMQP); (v) Data Distribution Service (DDS); (vi) OSGP; (vii) Open Platform Communication (OPC); and (viii) OPC Unified Architecture (UA). The two most common types of communication in IoT are request/response, as in CoAP, and publish/subscribe, used in MQTT.

The MQTT protocol was introduced by IBM in 1999 and standardized by OASIS in 2013 [18]. This protocol provides built-in connectivity between applications, middleware, networks, and communication technology. MQTT is asynchronous, based on publish/subscribe communication, and is made up of a broker (who contains topics) and several clients (who publish or subscribe to the topics). A client can send data to a topic or receive data from a topic it subscribes as a publisher updates this topic. This protocol has reliability on three Quality of Service (QoS) levels: (i) fire and forget; (ii) delivered at least once; (iii) delivered exactly once. MQTT executes over TCP, and thus security is performed using the services of this protocol [18,19,20,21]. MQTT and RESTfull are nowadays the most widely accepted and supported communication protocols for IoT, but CoAP might as well establish itself as a messaging standard in the future [22].

**Figure 1:**
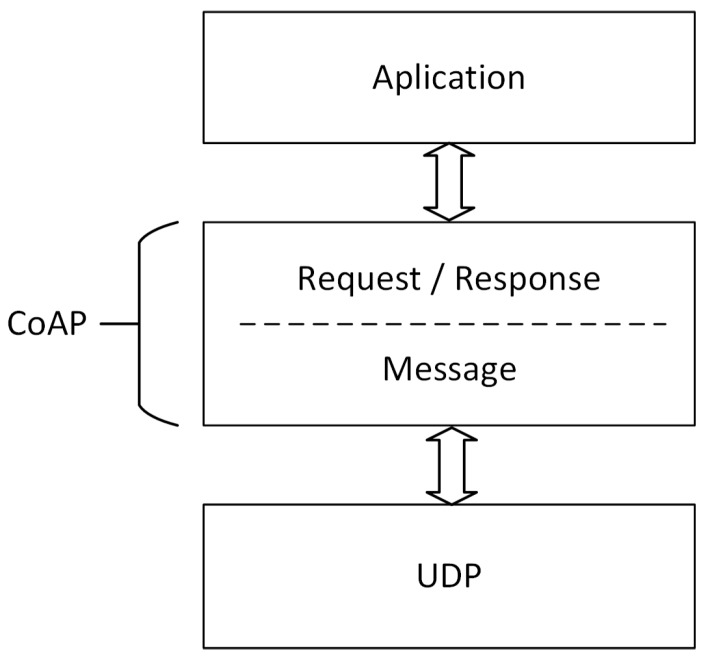
Simplified layered stack of Constrained Application Protocol (CoAP) (adapted from [23]).

CoAP is a synchronous request/response protocol developed for use with resource-constrained devices. It uses communication methods from Hypertext Transfer Protocol (HTTP), such as PUT, POST, GET, and DELETE, which allows these two protocols to work together [23]. CoAP allows interactions following a client/server architecture and has a lightweight implementation because it operates over User Datagram Protocol (UDP). The reliability mechanisms are implemented through two bits in the packet header, which define the message type and the QoS level. The messages can be: (i) commitable; (ii) unverifiable; (iii) recognition; and (iv) reset [17,18,20]. Figure 1 simply illustrates the protocol stack of CoAP by identifying its two layers.

### 2.3. Open Smart Grid Protocol

OSGP is a reference architecture for communication between devices operating over SGs [24]. The main purpose of this architecture is to provide greater control of electricity consumption and supply between customers and service providers in order to provide useful information to consumers of these services. OSGP is standardized by European Telecommunications Standards Institute (ETSI) and its layers are defined by the ETSI GS OSG 001, ISO/IEC 14908 and ETSI TS 103 908 specifications. OSGP is designed to work on a variety of SG devices. In order to avoid collisions, the protocol uses a master-slave architecture, and the nodes are not able to hear each other. As OSGP does not support overlapping transactions, the procedures must be executed rigorously one at a time.

**Figure 2:**
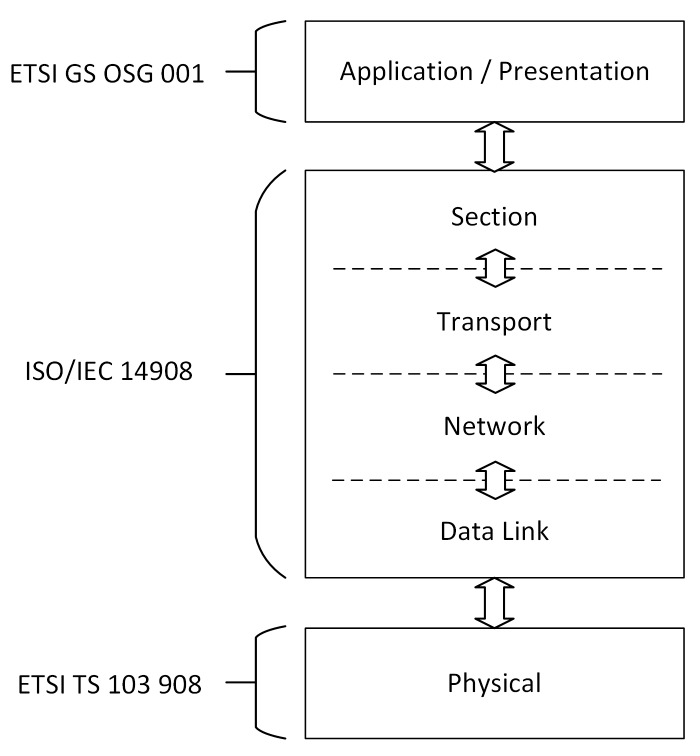
Simplified layered stack of Open Smart Grid Protocol (OSGP) (adapted from [27]).

OSGP is divided into three main layers: (i) Application (ETSI GS OSG 001) [24]; (ii) Network (ISO/IEC 14908); and (iii) Physical (ETSI TS 103 908). Figure 2 illustrates the protocol stack and the corresponding seven layers of the Open System Interconnection (OSI) model. The protocol is of request/response type [25,26].

## 3. Related Work

This section discusses a set of works that present the adaptation of CoAP to communication protocols used in SGs, such as the ETSI M2M, Distributed Network Protocol 3.0 (DNP3.0) and International Electrotechnical Commission (IEC) 61850 protocols.

As we can see in the works summarized in Table 1, some techniques used for protocol adaptation include native Application Interface Programming (API), Uniform Resource Identifier (URI), and packet mapping. These solutions are analyzed below.

In [28], the authors present the ETSI M2M system that addresses some issues in SGs identified and discussed in the literature. The work adopts CoAP as its native application layer protocol and uses it to carry all messages. Such services are made available using an open and native API. CoAP is used for communication with the transmission system, the distribution system, and consumers. The service-oriented demonstration prototype enables integration of a variety of M2M devices and utilizes a variety of communication technologies such as IEEE 802.15.4, 3G and GSM/GPRS.

The authors of [29] present an integration between CoAP and DNP3.0 protocols in order to implement a gateway to support CoAP in SGs; the integration allows communication M2M communication. To perform the integration between the protocols, the authors implemented a mapping layer as an interface. This interface is required because both protocols use layers that offer services with different protocols. DNP3.0 uses High-level Data Link Control (HDLC) and CoAP uses UDP. The authors used the GET and PUT methods of CoAP mapped, respectively, to the READ and WRITE methods of DNP3.0 to test and evaluate their implementation. A comparison of CoAP with Simple Object Access Protocol (SOAP) showed that the former performs better than the latter because SOAP adopts TCP, even using DNP3.0 services through adaptations.

An integration of CoAP with the IEC 61850 protocol for SGs is presented in [30]. In the paper, the authors also applied a mapping layer approach to tailor message exchange between CoAP and IEC 61850, which uses TCP as the transport layer. The methods offered by both protocols are mapped to perform the conversion and enable message exchange. A gateway is responsible for protocol integration through a mapping layer. On conversion, the IEC 61850 protocol information model is converted to a URI of the CoAP protocol. In traffic tests, the authors observed that packet delay in the network was negatively affected, having a higher latency than in the individual evaluation of the protocols.

In [31], the authors review protocols possible to be integrated with IEC 61850. Through this review, the authors decided to use CoAP because it is a standard already used in IoT and for being targeted at resource-constrained devices. The mapping of CoAP (GET, PUT, POST, and DELETE) methods to IEC 61850 methods is performed using CoAP URI. For adaptation between the protocols, the authors developed modules that have specific functions and reduce the complexity of integration. The authors state that their implementation is better than that of [30] because it covers all Abstract Communication Service Interface (ACSI) services. To improve the adaptation between the protocols, in [32], the same authors developed a version integrating CoAP and Concise Binary Object Representation (CBOR) in which they reduced the message size by 44%. This result was possible because CBOR requires fewer bytes than JavaScript Object Notation (JSON) and Extensible Markup Language (XML) and CoAP requires fewer bytes on the network than HTTP.

Likewise, the authors of [33] also used the IEC 61850 protocol for SGs, but adopted XMPP for communicating with devices. Besides, the authors took a different approach to the integration between protocols using the packet mapping method.

The works described above demonstrate the relevance of adapting CoAP for use in conjunction with SGs systems. According to the reported literature, the integration of a widely used IoT protocol developed for resource-constrained devices allows increasing the insertion of devices into the system. This integration enables a broader range of functionalities to be made available to SGs, as well as adding higher levels of management and automation to consumers and service providers.

As observed in the literature, most studies present solutions oriented to the IEC 61850 protocol using URI mapping to adapt this protocol with an IoT protocol. In this article, we propose a solution based on packet mapping to integrate CoAP with OSGP, which is a reference architecture for communication between devices operating over SGs. It is worth noting that the work [33] also applies the packet mapping method employed in this work. This method enables extracting data and addresses from OSGP packets to adapt them to CoAP and vice versa. This OSGP–CoAP mapping is different from the ones presented in the other works, with the vast majority focusing on the CoAP and IEC 61850 protocols.

## 4. Architecture

The solution proposed in this work is named COIIoT, the acronym for “CoAP and OSGP Integration for Internet of Things”. This section presents the architecture of COIIoT, including the algorithms that describe its mapping methods.

**Figure 3:**
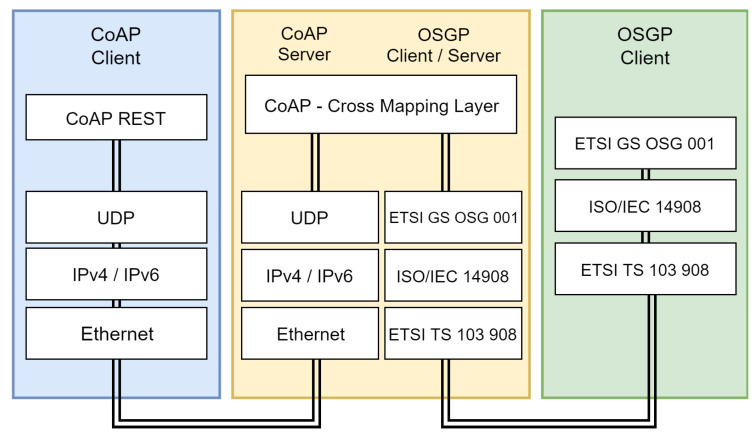
COIIoT architecture.

We employed the default frameworks of CoAP and OSGP without any modification. For the integration between the protocols, we applied a function mapping layer, individually assigning each type of request/response. We defined the mapping between packets so that the OSGP packet travels internally in CoAP and vice-versa. The proposed model acts as a gateway, serving for the integration between the protocols. Its structure is shown in Figure 3. CoAP uses UDP on the transport layer to travel to the gateway, while OSGP travels through its specific protocols to SGs, i.e., ISO/IEC 14908, and ETSI TS 103 908.

We also addressed the mapping of requests and responses and used two packet types in OSGP: request (which is categorized as Full or Partial) and response. For CoAP, we implemented the PUT, GET, and POST methods and use the MicroCoAP [34] library for implementation. This library was chosen because it has a small size and comprises all the methods necessary for this work. The entire development was done using C language to enable future implementations on resource-constrained devices such as microcontrollers.

As specified by the application layer [24], the OSGP transmission packet has all the message fields in Most Significant Byte (MSB) format, with the data field being the only exception. For security reasons, all the messages carry a sequence number and a summary. For pending tables, the Pending Event Descriptor (PED) is included in all count and length fields when this information is present. The current version of COIIoT does not yet fully implement the support for pending tables, as it is not a requirement for communication. However, this version already provides the treatment of this structure in code if it is requested.

The checksum is calculated based on the PED and data fields. The offset is not influenced by the presence of the PED in partial reads and writes. For instance, to read four bytes of offset three from a pending table, count would equal 10, and offset would equal 3 [35]. Through the table ID, the device using OSGP knows whether it must wait for the PED or not.

**Figure 4:**
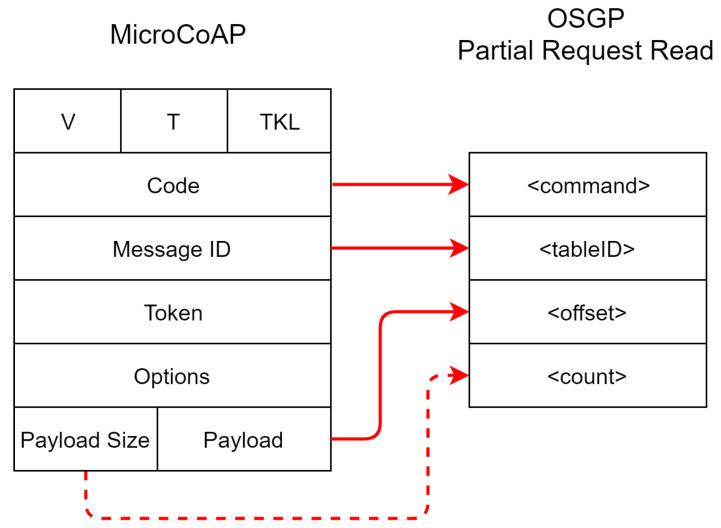
Mapping a request packet from CoAP to OSGP.

Figure 4 shows the packet mapping between CoAP and OSGP. In this figure, the client CoAP sends a message directed to the SG network via COIIoT) interface. CoAP executes a GET method using the MicroCoAP library, which is mapped to an OSGP Partial Read request. At the mapping, the CoAP-based packet extracts the request type, the message identifier, and the packet size, and then analyzes the received message. Afterward, the mapping layer matches the request received from CoAP to a request to be sent to OSGP. The payload of the CoAP packet is mapped into two fields of the OSGP packet. The first field (count) contains the message size, and the second field (offset) encloses the contents of the message.

**Figure 5:**
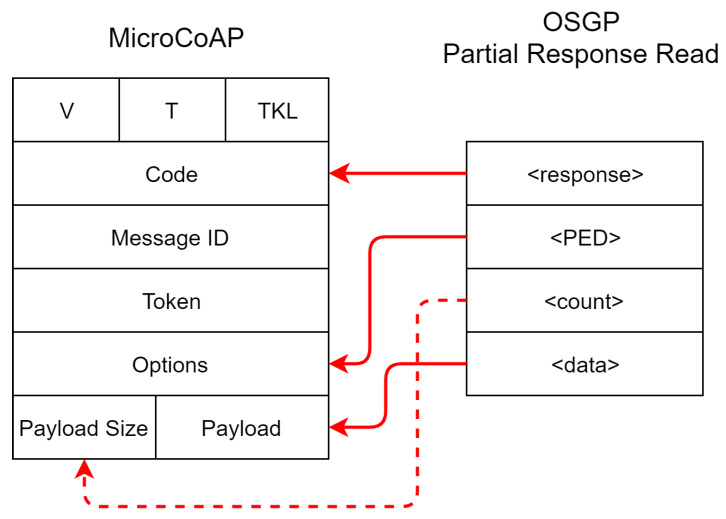
Mapping a response packet from OSGP to CoAP.

In Figure 5, the OSGP packet inserted into PED for pending queue management is shown; it will be included in the options field of CoAP. The count field reports the message size and is mapped directly to the payload size field of the CoAP packet. Among the services mapped between CoAP and OSGP, some of them depend on information from other protocols and implementations, such as pending events from OSGP.

**Figure 6:**
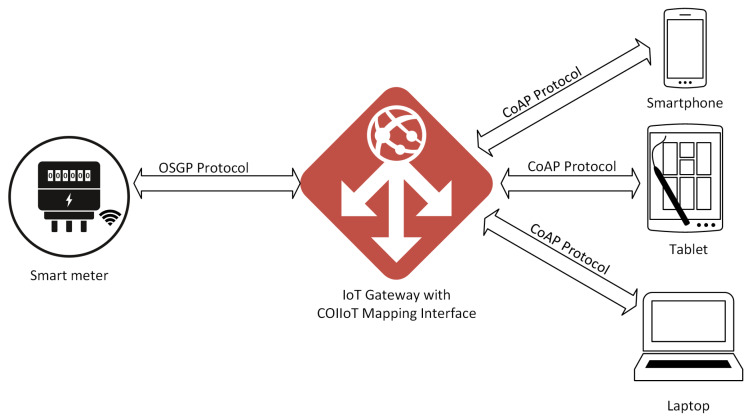
Example of a COIIoT-based system.

Figure 6 illustrates the application context of the COIIoT interface. The access to the information present in OSGP-based smart meters can be done utilizing devices that use CoAP to control the information transmitted in a smart home.

Algorithms 1–4 describe the methods used to map requests from the CoAP domain to the OSGP domain. It is worth noting that in the OSGP domain, a packet is called Application Protocol Data Unit (APDU). In Algorithm 1, the tableID parameter, which is as an identifier in OSGP, takes the protocol header identifier from CoAP. The command that will be executed is defined by code and remains unchanged. The mapping still checks against PED because if the OSGP packet implements this attribute, it is mapped to a CoAP parameter. Algorithm 2 differs from the first algorithm because it writes in the full table, while Algorithm 1 use an offset (line 4) to select a region of the table. Algorithms 3 and 4 perform partial and full reads from a table, respectively. The mappings of fields are similar to the ones performed in the first two algorithms.
**Algorithm 1:** PUT_to_Partial_Write_request
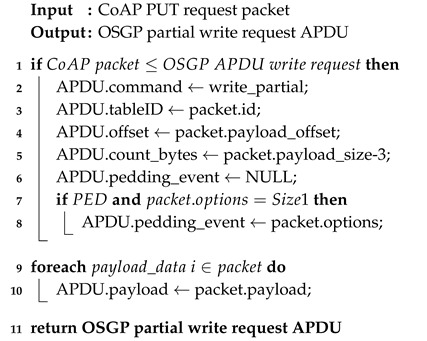


**Algorithm 2:** POST_to_Full_Write_request

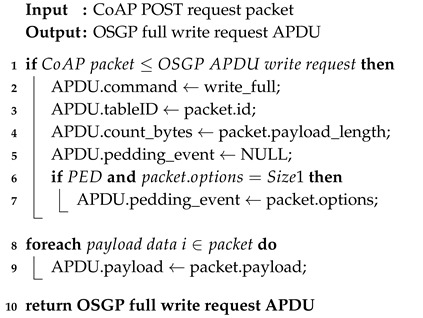



**Algorithm 3:** GET_to_Partial_Read_request

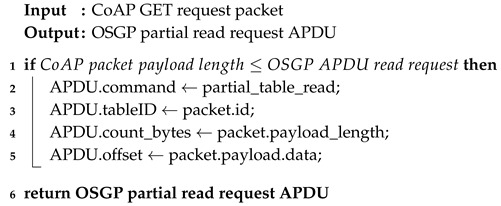



**Algorithm 4:** GET_to_Full_Read_request  **Input**   :  CoAP GET request packet  **Output**:  OSGP full read request APDU **1** APDU.tableID ← packet.id; **2** APDU.command ← full_table_read; **3** **return OSGP full read request APDU**

**Figure 7:**
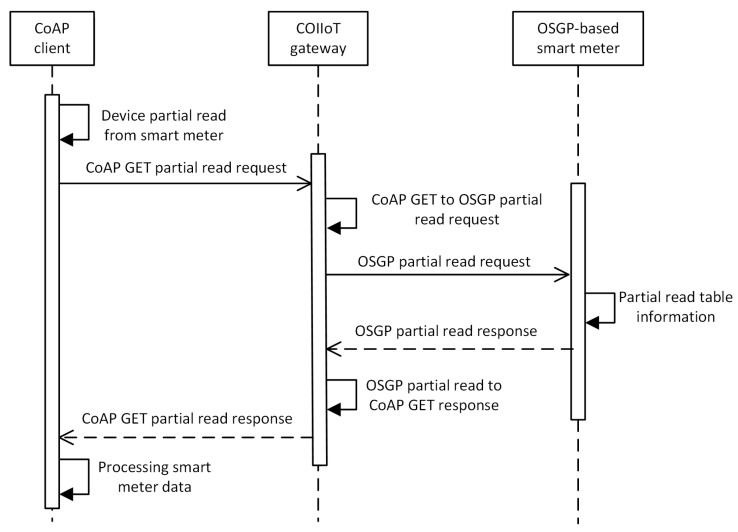
Sequence diagram for a partial read from the smart meter.

Algorithms A1–A4 (shown in Appendix A) describe the methods developed to map requests from the OSGP domain to the CoAP domain, while Algorithms A5–A8 (also shown in Appendix A) describe the methods designed to map the responses to the requests received by each domain. It is worth noting that the same method is used to map the responses for partial and full write requests, as well as for partial and full read requests. Figure 7 illustrates the use of the methods described in Algorithms 3 and A6 for an application environment using the ESP32 board as the gateway platform.

We can see from the algorithms that packets from both protocols have their fields redistributed to the other protocol during mapping. This approach differs from the one used in the works mentioned above, which apply URI mapping.

It is worth mentioning that the algorithms presented above (and in Appendix A) do not represent all the constraints imposed by the programming language and the libraries used, which mainly add complexity in the implementation.

## 5. Experimental Results

This section describes the materials and methods used in the development of the integration interface between CoAP and OSGP and presents and discusses the experimental results.

### 5.1. Evaluation Platforms

To evaluate the proposed solution, we developed two experimental platforms. On the first platform (named PC-based), we implemented a gateway using a desktop computer and emulated two different CoAP clients, one using the Copper plug-in of the Mozilla Firefox browser and another client implemented using the libCoAP library. The test environment that simulated the gateway featured the Debian GNU/Linux 8 (Jessie) 64-bit operating system, Intel^®^ Dual-Core^TM^ 2.5 GHz 64-bit processor and 4 GB of main memory. In testing, we measured the time to perform mapping on each communication, and verified and characterized function by function. For testing, we used varying OSGP packet sizes up to 114 bytes (the limit specified by the protocol). For CoAP, we adopted the same packet size in all communications, i.e., 512 bytes. Our goal with this experimental platform was to verify the proposed algorithms and validate the mapping layer using real CoAP clients.

In the second platform (named ESP-based), we implemented the gateway using the ESP32 development kit from Espressif Systems (the acronym ESP comes from the name of the company) [36]. ESP32 is a single 2.4 GHz Wi-Fi-and-Bluetooth combo chip designed with the TSMC ultra-low-power 40 nm technology. ESP32 is designed for mobile, wearable electronics, and IoT applications. It has a single or two Tensilica Xtensa 32-bit LX6 microprocessor cores with 448 KB ROM, 520 KB SRAM, and 16 KB SRAM. It also includes built-in antenna switches, low-noise receive amplifier, filters, power amplifier, and power-management modules. It is worth noting that ESP32 is open-source. The schematics and the printed circuit board layout are freely available to be used as a reference for developing fully-customized ESP32-based hardware designs.

Using the ESP32 platform, we emulated a scenario in which a CoAP client (e.g., a smartphone) sends requests to the gateway in order to obtain information from the OSGP domain, as well as responds messages sent by the emulated OSGP-based device (e.g., a smart meter). To carry out this evaluation, we used the Espressif IoT Development Framework (ESP-IDF) 3.3.1 environment. Our goal with this platform was to assess the performance of a low-cost and low-power embedded gateway and evaluate the feasibility of using the proposed solution in a real-life environment.

### 5.2. Results

Table 2 summarizes the results obtained from the experiments performed using the PC-based platform, presenting the memory requirements for every packet and the latency of each mapping function. We can observe that the OSGP packets require less memory than the CoAP packets, which has the same size for all the functions. It is worth mentioning that all the tests were performed using packets with a 6-byte payload. Regarding performance, we can observe that the most expensive communication is the one that comprises the mapping of a CoAP PUT request to an OSGP Partial Write request (790 ns), and its corresponding response (470 ns). This worst-case mapping consumes 1260 ms for request and response. The experimental results also show that the response algorithms, although relatively simple in their implementation, have a relatively high impact when compared to request methods. This cost is due in part to the library used to implement the CoAP protocol.

The low latency observed in the results above is due to the type of gateway used in our experiments, a desktop computer that plays the role of a server present in the environment automated by IoT technologies. This approach allows for a low interface impact in conjunction with other features that may be present in home automation systems, for example. However, it is worth noting that a PC-based gateway has high power consumption (e.g., 65 W in a laptop and 200 W in a desktop computer).

The ESP-based platform operated at 5 V and drained 52 mA to run the mapping functions, which results in power consumption of about 260 mW. On the other hand, the latency to process each function ranges from 8 to 30 μs as it is presented in Table 3.

### 5.3. Discussion

The experimental results demonstrate that using a low-power platform increases the processing latency significantly (up to two orders of magnitude). However, its impact is negligible when considering the requirements of most of the IoT applications and the typical latency of communication on the Internet. Therefore, the proposed solution enables to integrate the CoAP and OSGP protocols in a solution that presents low latency even when running on an embedded platform.

When comparing the solution proposed in this work with that of [33], mainly relating the protocols for SGs (i.e., OSGP and IEC 61850), the solution proposed in this work based on OSGP focuses on end-users (i.e., consumers), while the target audience of solutions such as [33] are energy substations [25]. Besides, integrating CoAP with an SG protocol aimed the end-user to restrict the information available to himself, only in his/her smart meter.

The solution presented in [28] does not indicate quantitative results of implementation that validate its API and the gateway that makes use of Graphical User Interface (GUI), which prevents the use in embedded systems with resource restrictions. On the other hand, solutions based on URI mapping require the construction of the URI, as indicated in [23], to carry out the building of the CoAP packet, for instance.

It is noteworthy to mention that our evaluations were performed on mapping methods only and did not take the function calls into account. This approach was used because this work describes a mapping layer that integrates a larger IoT system. Another aspect that we did not address in this work is the security of the transmitted data because it does not impact the interface operation directly. We consider that security is a requirement that can be addressed directly at the sender and receiver ends, which is beyond the scope of this work. Finally, it must be highlighted that the current version of COIIoT was evaluated on an experimental scenario for proof of concept. It still must be evaluated in a real scenario composed of a large number of smart meters.

## 6. Conclusions

This work presented COIIoT, a mapping interface for the integration between CoAP and OSGP. The goal of this interface is to enable the data exchange between IoT devices used in home and industry applications and an SG infrastructure. The developed interface comprises a set of mapping functions that translates the methods used in each protocol and has a low cost that enables its use with low impact in communication. Furthermore, the mapping interface reduces the time necessary for application development as it abstracts the complexity involved in the communication between the protocols.

It is noteworthy that while the technique used in this work maps packet directly to packet, other techniques for protocols integration result in an overhead of information and require the building of the URI, or even the use of a GUI, which restricts its use on platforms that have limited resources for storage and processing.

In future work, we intend to apply COIIoT in a physical testbed to evaluate the embedded gateway when interfacing a smartphone with a real smart meter within an SG infrastructure.

## Figures and Tables

**Table 1:** Related work.

Work	IoT	SG	Adaptation
[28]	CoAP	ETSI M2M	Native API
[29]	CoAP	DNP3.0	URI mapping
[30]	CoAP	IEC 61850	URI mapping
[31]	CoAP	IEC 61850	URI mapping
[32]	CoAP+CBOR	IEC 61850	URI mapping
[33]	XMPP	IEC 61850	Packet mapping
This work	CoAP	OSGP	Packet mapping

Note: CBOR – Concise Binary Object Representation.

**Table 2:** Packets size and latency of mapping methods running on the PC-based platform.

Type	Method	Algorithm	CoAP (Bytes)	OSGP (Bytes)	Latency (ns)
Request	PUT→Partial Write	1	512	112	790
Request	POST→Full Write	2	512	88	520
Request	GET→Partial Read	3	512	64	375
Request	GET→Full Read	4	512	24	275
Request	Partial Write→PUT	5	512	112	646
Request	Full Write→POST	6	512	88	390
Request	Partial Read→GET	7	512	64	196
Request	Full Read→GET	8	512	24	265
Response	Write→PUT/POST	9	512	8	470
Response	Read→GET	10	512	72	440
Response	PUT/POST→Write	11	512	8	286
Response	GET→Read	12	512	72	310

**Table 3:** Latency of mapping methods running on the ESP-based platform.

Type	Method	Algorithm	Latency (μs)
Request	PUT→Partial Write	1	30
Request	POST→Full Write	2	16
Request	GET→Partial Read	3	20
Request	GET→Full Read	4	8
Request	Partial Write→PUT	5	30
Request	Full Write→POST	6	29
Request	Partial Read→GET	7	30
Request	Full Read→GET	8	19
Response	Write→PUT/POST	9	9
Response	Read→GET	10	24
Response	PUT/POST→Write	11	9
Response	GET→Read	12	9

## Abbreviations

The following abbreviations are used in this manuscript:

6LoWPAN	IPv6 over Low power Wireless Personal Area Networks
ACSI	Abstract Communication Service Interface
AMI	Advanced Metering Infrastructure
AMR	Automatic Meter Reading
AMQP	Advanced Message Queuing Protocol
APDU	Application Protocol Data Unit
API	Application Interface Programming
CBOR	Concise Binary Object Representation
CoAP	Constrained Application Protocol
COIIoT	CoAP and OSGP Integration for Internet of Things
CPS	Cyber-Physical System
DDS	Data Distribution Service
DNP3.0	Distributed Network Protocol 3.0
EPC	Electronic Product Code
ETSI	European Telecommunications Standards Institute
GUI	Graphical User Interface
HDLC	High-level Data Link Control
HTTP	Hypertext Transfer Protocol
IEC	International Electrotechnical Commission

IETF	Internet Engineering Task Force
IoT	Internet of Things
IPv6	Internet Protocol v6
ITU	International Telecommunication Union
JSON	JavaScript Object Notation
M2M	Machine-to-Machine
MQTT	Message Queue Telemetry Transport
MSB	Most Significant Byte
OPC	Open Platform Communication
OPC UA	OPC Unified Architecture
OSGP	Open Smart Grid Protocol
OSI	Open System Interconnection
PED	Pending Event Descriptor
PMU	Phasor Measurement Unit
QoS	Quality of Service
RFID	Radio-Frequency Identification
RPL	Routing Protocol for Low Power and Lossy Networks
SG	Smart Grid
SOAP	Simple Object Access Protocol
TCP	Transmission Control Protocol
UDP	User Datagram Protocol
URI	Uniform Resource Identifier
WSN	Wireless Sensor Networks
XaaS	Everything-as-a-Service
XMPP	Extensible Messaging and Presence Protocol
XML	Extensible Markup Language

## Appendix A

This appendix present the algorithms designed for the methods implemented to map the requests from the OSGP domain to the CoAP domain (Algorithms A1–A4), as well as the responses between the two domains (Algorithms A5–A8).

Algorithms A1 and A2 map a partial and full write operations from the OSGP domain to PUT and POST operations from the CoAP domain, respectively. These algorithms use a structure similar to that used in the algorithms described in Section 4. It is important to mention that the mapping of a partial write operation (Algorithm A1) concatenates the offset with the payload (line 8).

**Algorithm A1:** Partial_Write_to_PUT_request
**Input** : OSGP partial write request APDU **Output**: CoAP PUT request packet **1** packet.version ← CoAP Version; **2** packet.request_type ← PUT; **3** packet.hdr.code ← WRITE; **4** packet.id ← APDU.tableID; **5** packet.command ← APDU.command; **6** packet.options ← 0; **7** packet.payload_length ← APDU.count_bytes; **8** packet.payload.data ← APDU.offset error APDU.payload; **9** **return CoAP PUT request packet**

**Algorithm A2:** Full_Write_to_POST_request
**Input** : OSGP full write request APDU **Output**: CoAP POST request packet **1** packet.version ← CoAP Version; **2** packet.request_type ← POST; **3** packet.hdr.code ← WRITE; **4** packet.id ← APDU.tableID; **5** packet.command ← APDU.command; **6** packet.options ← 0; **7** packet.payload_length ← APDU.count_bytes; **8** packet.payload.data ← APDU.payload; **9** **return CoAP POST request packet**

Algorithms A3 and A4 map read packets from OSGP to packets of CoAP which implement the GET method. As they deal with read operations, no data is loaded in the payload. However, in partial reads, it is necessary to inform the size of the offset.

**Algorithm A3:** Partial_Read_to_GET_request
**Input** : OSGP partial read request APDU **Output**: CoAP GET request packet **1** packet.version ← CoAP Version; **2** packet.request_type ← GET; **3** packet.hdr.code ← READ; **4** packet.id ← APDU.tableID; **5** packet.command ← APDU.command; **6** packet.options ← 0; **7** packet.payload_length ← offset_size; **8** packet.payload.data ← APDU.offset; **9** **return CoAP GET request packet**

**Algorithm A4:** Full_Read_to_GET_request
**Input** : OSGP full read request APDU **Output**: CoAP GET request packet **1** packet.version ← CoAP Version; **2** packet.request_type ← GET; **3** packet.hdr.code ← READ; **4** packet.id ← APDU.tableID; **5** packet.command ← APDU.command; **6** packet.options ← 0; **7** packet.payload.data ← NULL; **8** **return CoAP GET request packet**

Algorithms A5–A8 map the responses to the requests described in the previous algorithms. It is worth noting that a single method is used to map a response to the different variations of requests of the same operation (partial/full or PUT/POST). Therefore, we have defined only four response packet mapping algorithms. Since the response packets to write operations only notify about the success of the operation, their mapping algorithms are very concise. On the other hand, the mapping algorithms for the response to read operations should also include the data read.

**Algorithm A5:** Write_to_PUT_POST_response

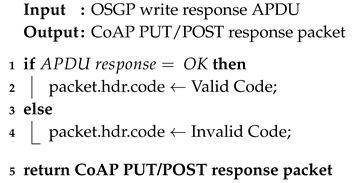

**Algorithm A6:** Read_to_GET_response

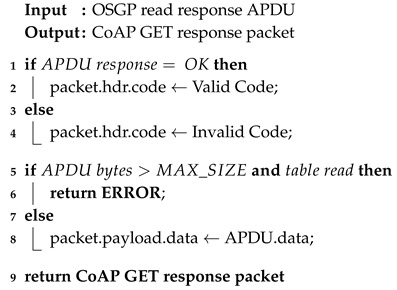

**Algorithm A7:** PUT_POST_to_Write_response

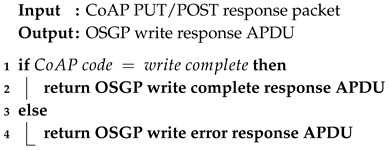

**Algorithm A8:** GET_to_Read_response
